# Synergizing traditional CT imaging with radiomics: a novel model for preoperative diagnosis of gastric neuroendocrine and mixed adenoneuroendocrine carcinoma

**DOI:** 10.3389/fonc.2024.1480466

**Published:** 2024-10-23

**Authors:** Xiaoxiao He, Sujun Yang, Jialiang Ren, Ning Wang, Min Li, Yang You, Yang Li, Yu Li, Gaofeng Shi, Li Yang

**Affiliations:** ^1^ Department of Computed Tomography and Magnetic Resonance, Fourth Hospital of Hebei Medical University, Shijiazhuang, Hebei, China; ^2^ Department of Computed Tomography and Magnetic Resonance, Handan Central Hospital, Handan, Hebei, China; ^3^ Department of Pharmaceuticals Diagnostics, GE HealthCare, Beijing, China; ^4^ Department of Computed Tomography, Zhengding Country People’s Hospital, Shijiazhuang, Hebei, China

**Keywords:** gastric carcinoma, neuroendocrine carcinoma, mixed adenoneuroendocrine carcinoma, traditional X-ray computed tomography, radiomics

## Abstract

**Objective:**

To develop diagnostic models for differentiating gastric neuroendocrine carcinoma (g-NEC) and gastric mixed adeno-neuroendocrine carcinoma (g-MANEC) from gastric adenocarcinoma (g-ADC) based on traditional contrast enhanced CT imaging features and radiomics features.

**Methods:**

We retrospectively analyzed 90 g-(MA)NEC (g-MANEC and g-NEC) patients matched 1:1 by T-stage with 90 g-ADC patients. Traditional CT features were analyzed using univariable and multivariable logistic regression. Tumor segmentation and radiomics features extraction were performed with Slicer and PyRadiomics. Feature selection was conducted through univariable analysis, correlation analysis, LASSO, and multivariable stepwise logistic. The combined model incorporated clinical and radiomics predictors. Diagnostic performance was assessed with ROC curves and DeLong’s test. The models’ diagnostic efficacy was further validated in subgroup of g-NEC vs. g-ADC and g-MANEC vs. g-ADC cases.

**Results:**

Tumor necrosis and lymph node metastasis were independent predictors for differentiating g-(MA)NEC from g-ADC (*P* < 0.05). The clinical model’s AUC was 0.700 (training) and 0.667(validation). Five radiomics features were retained, with the radiomics model showing AUC of 0.809 (training) and 0.802 (validation). The combined model’s AUCs were 0.853 (training) and 0.812 (validation), significantly outperforming the clinical model (*P* < 0.05). Subgroup analysis revealed that the combined model exhibited acceptable performance in differentiating g-NEC from g-ADC and g-MANEC from g-ADC, with AUC of 0.887 and 0.823 in the training cohort and 0.852 and 0.762 in the validation cohort.

**Conclusion:**

A combined model based on traditional CT imaging and radiomic features provides a non-invasive and effective preoperative diagnostic method for differentiating g-(MA)NEC from g-ADC.

## Introduction

Gastric neuroendocrine tumors represent a highly heterogeneous group of tumors originating from neuroendocrine cells and peptidergic neurons. Gastric neuroendocrine carcinoma (g-NEC) and gastric mixed adenocarcinoma-neuroendocrine carcinoma (g-MANEC) are both poorly differentiated neuroendocrine tumors, accounting for approximately 0.4-0.6% of all malignant gastric epithelial tumors (1). The biological behavior of g-(MA)NEC differs from that of the more commonly encountered gastric adenocarcinoma (g-ADC). The former exhibits higher aggressiveness, with a greater tendency for early lymphatic and hematogenous spread, leading to a poorer prognosis (2, 3). Treatment approaches also differ between these tumors. The first-line treatment for g-(MA)NEC typically involves an etoposide and cisplatin (EP) regimen, whereas g-ADC is more commonly treated with a capecitabine and oxaliplatin (XELOX) regimen or a tegafur, gimeracil, and oteracil potassium combined with oxaliplatin (SOX) regimen (4). In terms of surgical techniques, procedures for g-(MA)NEC generally follow those used for g-ADC. However, there remains controversy regarding the extent of lymph node dissection and the use of neoadjuvant therapy (5–8). Therefore, accurate preoperative differentiation between g-(MA)NEC and g-ADC can aid in selecting appropriate treatment strategies and assessing patient prognosis.

Currently, the differentiation between g-(MA)NEC and g-ADC primarily relies on pathological examinations and immunohistochemical analyses (9). However, the limited tissue samples obtained from preoperative gastroscopic biopsies often lead to misdiagnoses, with many cases of g-NEC and most cases of g-MANEC being incorrectly identified as poorly differentiated g-ADC (10, 11). Postoperative pathology, although definitive, offers results that are too delayed to influence the choice of preoperative treatments and surgical approaches effectively. Computed Tomography (CT) is a commonly used imaging method for evaluating gastric tumors (12). Nevertheless, traditional CT imaging features of g-(MA)NEC and g-ADC are similar, making differentiation challenging. Radiomics, an emerging field, enables the extraction of quantitative features from images. By analyzing the distribution and spatial relationships of pixel intensities within an image, radiomics reflects tumor heterogeneity and detects subtle differences that are not visible to the naked eye. There have been studies reporting the application of radiomics in diagnosing gastric tumors, staging, and predicting pathological features (13–15).

In this study, we conduct a retrospective analysis of the clinical characteristics, traditional CT imaging features, and radiomic features of patients with surgically confirmed g-(MA)NEC. We explore the value of radiomics based on enhanced CT in distinguishing g-(MA)NEC from g-ADC. This approach not only aims to improve the accuracy of preoperative diagnoses but also strives to refine the decision-making process for treatment strategies, potentially leading to more personalized and effective management of gastric tumors.

## Materials and method

### Patient

**Figure 1:**
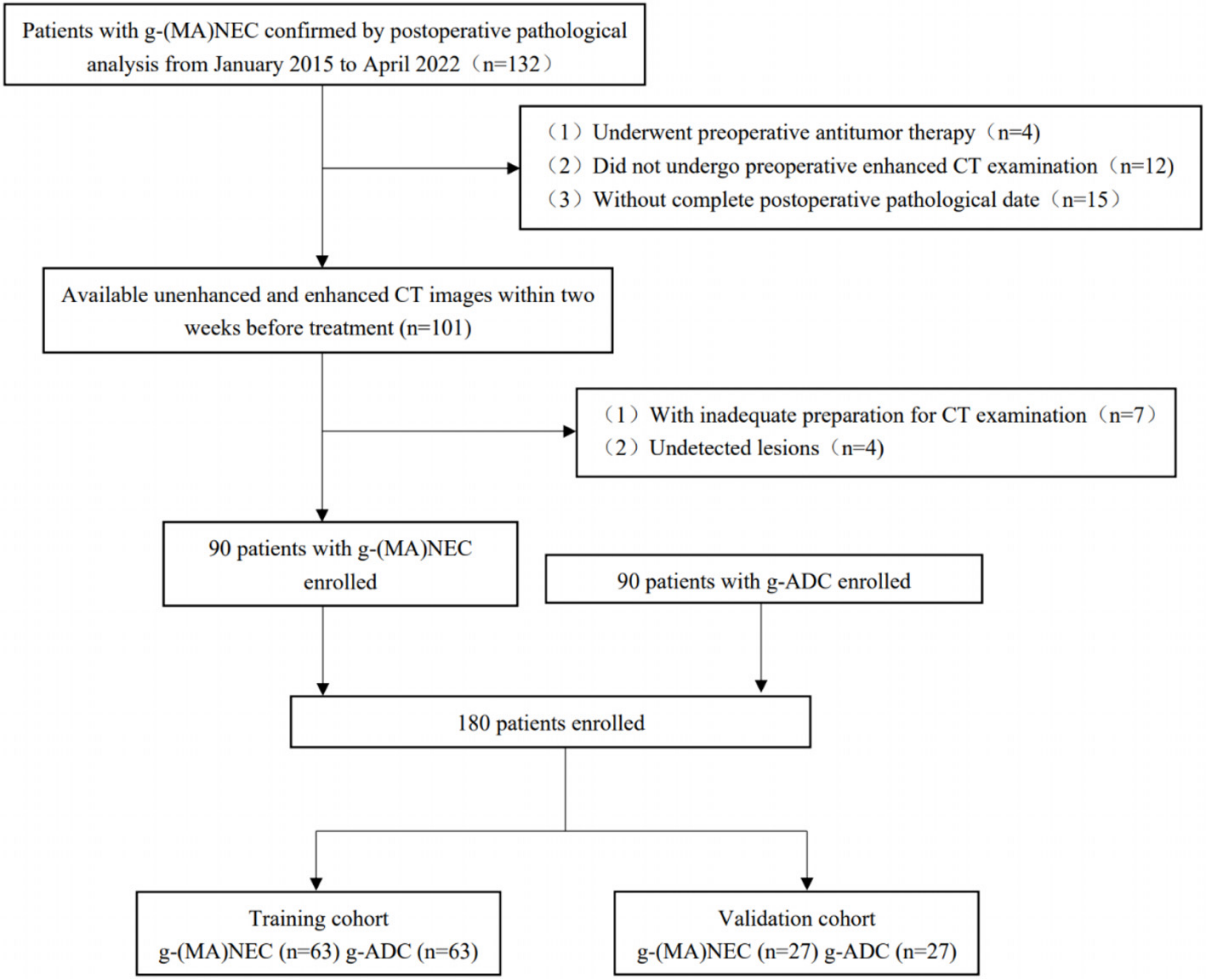
Flowchart of patient enrollment.

A retrospective analysis was conducted on the medical records of patients with g-(MA)NEC who were treated at the Fourth Hospital of Hebei Medical University from January 2015 to April 2022. Inclusion criteria: (1) patients who underwent surgical resection without receiving any anti-tumor treatments preoperatively, and whose postoperative pathology confirmed g-NEC or g-MANEC with complete pathological data; (2) patients who underwent abdominal and pelvic enhanced CT scans within two weeks prior to surgery with complete imaging data. Exclusion criteria: (1) poor gastric filling, peristaltic artifacts, and residual stomach contents that impaired tumor visualization; (2) tumors that were too small to be detected on imaging. Flowchart of patient enrollment in **Figure 1**.

### Image acquisition and preprocessing

Prior to their CT scans, patients were required to fast for 6-8 hours. Ten minutes before scanning, they were administered an intramuscular injection of 10 mg of scopolamine (Hangzhou Minsheng Pharmaceutical Industry, China) to reduce gastrointestinal motility. Additionally, patients were instructed to ingest 800-1000 ml of warm water to adequately distend the stomach. The CT scans were performed using the Revolution CT (GE Healthcare, USA) and the SOMATOM Definition Flash CT (Siemens Healthcare, Germany). The scanning range covered the area from the diaphragmatic dome to the pubic symphysis. The main scanning parameters of the two scanners were as follows: tube voltage of 120 kV, automatic tube current, rotation time of 0.5s, matrix of 512×512, slice thickness of 5mm, and interlayer spacing of 5mm. Non-ionic contrast agent (Iohexol, 300 mg/dL; GE Healthcare, USA) was injected intravenously through the elbow vein at a flow rate of 3 ml/s (2Ml/kg body weight). The enhanced CT images of arterial phase and venous phase were acquired at 35 s and 70 s after the injection of contrast agent, respectively.

### Clinical features analysis

Patient demographics such as gender and age were recorded. Two radiologists with extensive experience in abdominal imaging, with 5 years (XXH), and 19 years (LY) analyzed the conventional CT imaging features independently, without knowledge of the postoperative pathology. Discrepancies in their assessments were resolved through discussion until consensus was reached. The conventional CT imaging features evaluated included: the location, shape, and size of the primary tumor; the presence or absence of ulceration, mucosal coverage, and necrosis; enhancement patterns; CT attenuation values across different phases; normalized tumor enhancement ratio (NTER); and net enhancement values (△CT) across different phases. The presence of lymph node metastasis was also assessed. Additional, mucosal coverage refers to the tumor surface exhibiting a complete or partial mucosal layer, or symmetric mucosal elevation at both ends of the tumor (16). CT attenuation values were measured by selecting the largest cross-section of the tumor on 5 mm thick axial images, avoiding necrotic areas and surrounding gastric tissues. A circular region of interest (ROI) was delineated to measure the attenuation values of the solid components of the tumor. The NTER was calculated as $NTER=\frac{CT\;tumor\;value}{CT\;aortic\;value}\times 100\%$ , and △CT was determined by subtracting the non-enhanced CT value from the enhanced CT value ΔCT=CT enahnced value−CT non enhanced value. Lymph nodes meeting at least one of the following criteria were defined as metastatic (17, 18): (1) short axis greater than 1 cm or a short-to-long axis ratio greater than 0.7; (2) high enhancement or heterogeneous enhancement; (3) lymph nodes clustered in a drainage area. Univariable and multivariable logistic regression analyses were performed on these clinical and traditional CT imaging features to identify independent predictors capable of distinguishing g-(MA)NEC from g-ADC, leading to the development of a clinical model.

### Radiomics features analysis

**Figure 2:**
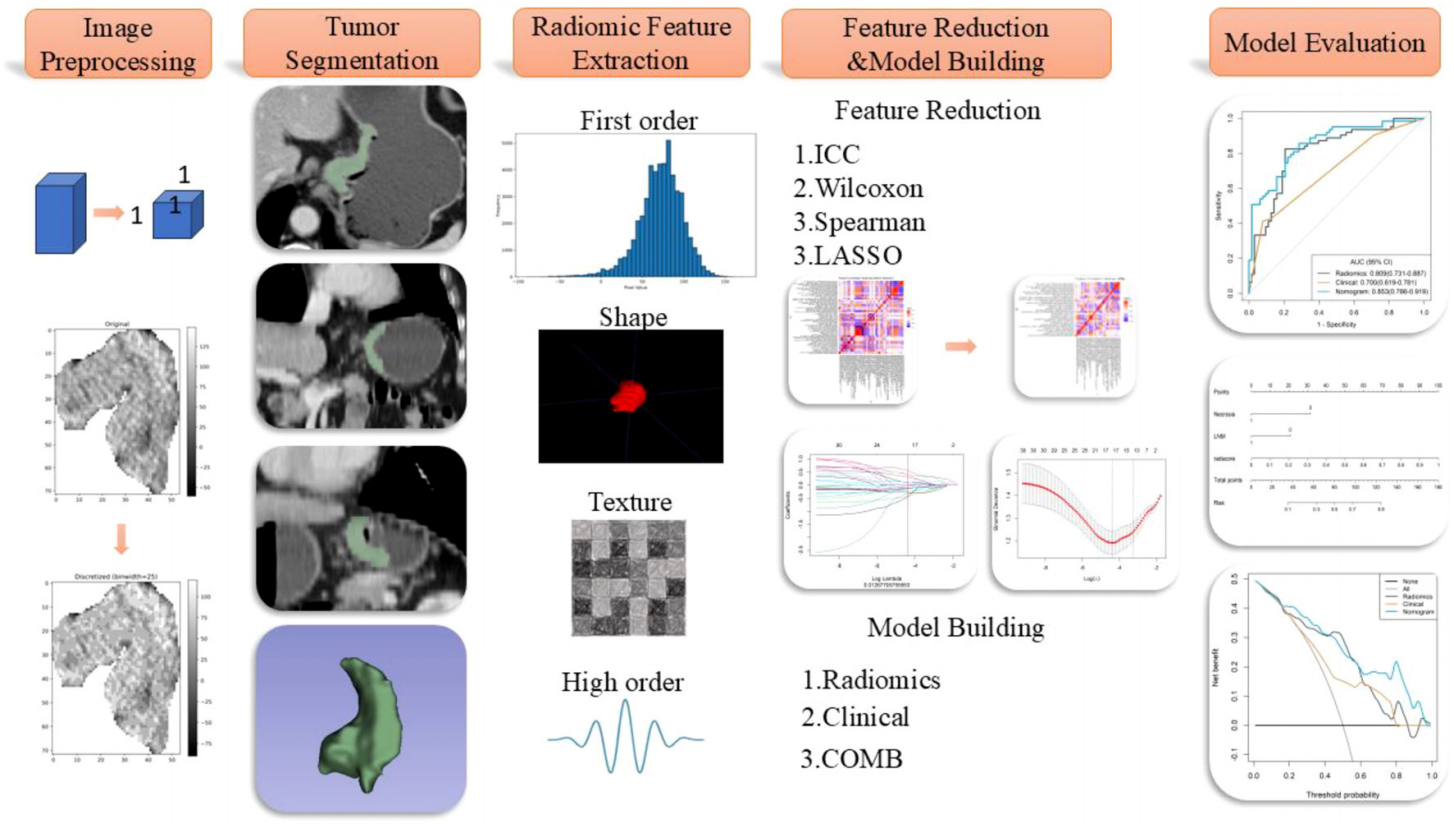
Schematic diagram of the radiomics workflow.

The radiomics workflow diagram of this study is presented in **Figure 2**. Tumor segmentation was meticulously performed using 3D Slicer software (version 5.2.2, https://www.slicer.org). The junior radiologist (XXH) manually delineated the region of interest (ROI) along the tumor edges on axial images with a thickness of 5mm during the venous phase, which was reviewed and confirmed by the senior radiologist (LY) to ensure precision in defining the tumor boundaries, essential for accurate subsequent analyses. To account for variations in scanning equipment that could affect voxel size and impact radiomic features, images were standardized by resampling them to a uniform resolution of 1×1×1 mm³. Additionally, image grayscale values were discretized into 25 levels to reduce the sparsity of the grayscale matrix, facilitating the computation of texture features. The preprocessed images underwent various transformations, including Laplacian of Gaussian filtering, wavelet transforms, gradient filtering, logarithmic filtering, and local binary pattern transformations to extract high-dimensional features critical for detailed texture analysis. Parameters for the Laplacian of Gaussian filters were set at LoG σ=5,7, with wavelet transformations incorporating eight filter parameters, and local binary patterns including three parameters.

Feature extraction was then conducted using the PyRadiomics platform (https://pyradiomics.readthedocs.io/). To prevent model overfitting and reduce redundancy in high-dimensional features, a robust methodology was employed. Firstly, a month after the initial ROI delineation, a random subset of 50 patients underwent a second ROI delineation to ensure consistency, retaining features with an Intraclass Correlation Coefficient (ICC) greater than 0.8. Univariable analysis (Mann-Whitney U test) was employed to retain features with P-values less than 0.05, and correlation analysis was used to remove features with a correlation coefficient greater than 0.9, ensuring feature independence. Finally, LASSO regression and multivariable stepwise regression were conducted to preserve features with independent predictive power. LASSO performs both feature selection and regularization, effectively handling multicollinearity and producing more generalizable models. The selected radiomic features were used to construct a radiomics model, culminating in the generation of a Radiomics Score (Rad-score). This score quantitatively encapsulates the tumor characteristics derived from the imaging data, providing a robust tool for clinical assessment and decision-making, thus enhancing the predictive accuracy of the model and ensuring its applicability across different patient populations by minimizing potential biases associated with image processing and feature extraction techniques. The independent predictive factors identified in the clinical model along with the Radiomics Score (Rad-score) outputted by the radiomic model were analyzed using multivariable stepwise logistic regression to generate combined model.

### Statistical analysis

Statistical analyses were performed using R software (version 3.8). The Shapiro-Wilks test was employed to assess the normality of continuous variables. For variables that followed a normal distribution, the independent samples t-test was utilized, whereas the Mann-Whitney U test was used for variables that did not exhibit normal distribution. Categorical variables were analyzed using either Fisher’s exact test or the chi-square test, depending on the data size and distribution, with a significance level set at *P* < 0.05. The diagnostic performance of the clinical model, radiomic model, and combined model was evaluated using Receiver Operating Characteristic (ROC) curves. The Area Under the Curve (AUC), accuracy (ACC), sensitivity (SEN), and specificity (SPE) were calculated to assess each model’s effectiveness. The DeLong test was applied to compare the AUC of the three models, providing a statistical basis for evaluating the superiority of one model over the others in differentiating between tumor types. Model calibration was examined using calibration curves, and the goodness-of-fit for each model was assessed with the Hosmer-Lemeshow test. Decision Curve Analysis (DCA) was performed to evaluate and compare the clinical usefulness of the three models.

## Result

### Patient characteristics

A total of 44 cases of g-NEC and 46 cases of g-MANEC met the inclusion and exclusion criteria. Postoperative pathological T staging identified 9 cases at T2, 78 at T4a, and 3 at T4b. These cases were matched 1:1 by pathological T stage with 90 cases of gastric adenocarcinoma that were also confirmed postoperatively and met the inclusion and exclusion criteria. The study ultimately included 180 patients, who were randomly divided into a training set (n=126) and a validation set (n=54) as shown in **Figure 1**.

### Clinical model evaluation

The distribution of clinical and traditional CT imaging features within the training cohort of patients is detailed in **Table 1**. Univariable analysis of this cohort revealed statistically significant differences between g-(MA)NEC and g-ADC in terms of tumor location, thickness, presence of necrosis, △CTvp and the presence of lymph node metastasis (*P* < 0.05). Further multivariable logistic regression analysis identified the presence of necrosis and lymph node metastasis as independent predictive factors for differentiating g-(MA)NEC from g-ADC (*P*<0.05). Based on these findings, a clinical model was constructed. The performance of this model in the training cohort and validation cohort demonstrated AUC of 0.700 (95% CI, 0.619-0.781) and 0.667 (95% CI, 0.533-0.800), respectively.

**Table 1:** Clinical and CT features of the patients in the training cohort.

Feature	(MA)NEC (n=63)	ADC (n=63)	*P*
**Gender**			0.180
Female	9 (14.3%)	16 (25.4%)	
Male	54 (85.7%)	47 (74.6%)	
**Age**	65.0 [59.0;70.0]	63.0 [56.0;69.0]	0.198
**Location**			<0.001
Upper	49 (77.8%)	19 (30.2%)	
Middle	9 (14.3%)	7 (11.1%)	
Lower	5 (7.9%)	37 (58.7%)	
**Borrmann**			0.273
Localized type	28 (44.4%)	21 (33.3%)	
Diffuse type	35 (55.6%)	42 (66.7%)	
**Length**	4.7 [3.3;5.8]	4.6 [3.6;5.5]	0.944
**Length Grade**			1.000
<5cm	38 (60.3%)	39 (61.9%)	
≥5cm	25 (39.7%)	24 (38.1%)	
**Thick**	2.1 [1.5;2.3]	1.7 [1.4;2.1]	0.021
**Thick Grade**			0.152
<2cm	30 (47.6%)	39 (61.9%)	
≥2cm	33 (52.4%)	24 (38.1%)	
**Mucosal coverage**			1.000
Negative	42 (66.7%)	42 (66.7%)	
Positive	21 (33.3%)	21 (33.3%)	
**Necrosis**			0.005
Negative	42 (66.7%)	56 (88.9%)	
Positive	21 (33.3%)	7 (11.1%)	
**Ulcer**			1.000
Negative	1 (1.6%)	2 (3.2%)	
Positive	62 (98.4%)	61 (96.8%)	
**Enhancement pattern**			0.099
Homogeneous	52 (82.5%)	59 (93.7%)	
Heterogeneous	11 (17.5%)	4 (6.3%)	
**CT_NON_**	40.7 [37.8;46.1]	40.8 [37.1;44.4]	0.620
**CT_AP_**	68.7[59.8;89.2]	74.7[60.7;96.2]	0.146
**CT_VP_**	85.6 [75.9;100.3]	92.2 [81.9;105.3]	0.056
**NTER_AP_**	0.3 [0.3;0.3]	0.3 [0.3;0.4]	0.243
**NTER_VP_**	0.6 [0.6;0.7]	0.7 [0.6;0.8]	0.121
**△CT_AP_**	29.0 [18.9;48.1]	38.0 [22.8;54.0]	0.111
**△CT_VP_**	43.9 [35.5;60.2]	53.9 [39.9;63.2]	0.037
**cN**			<0.001
Negative	8 (12.7%)	27 (42.9%)	
Positive	55 (87.3%)	36 (57.1%)	

### Radiomics model evaluation

**Figure 3:**
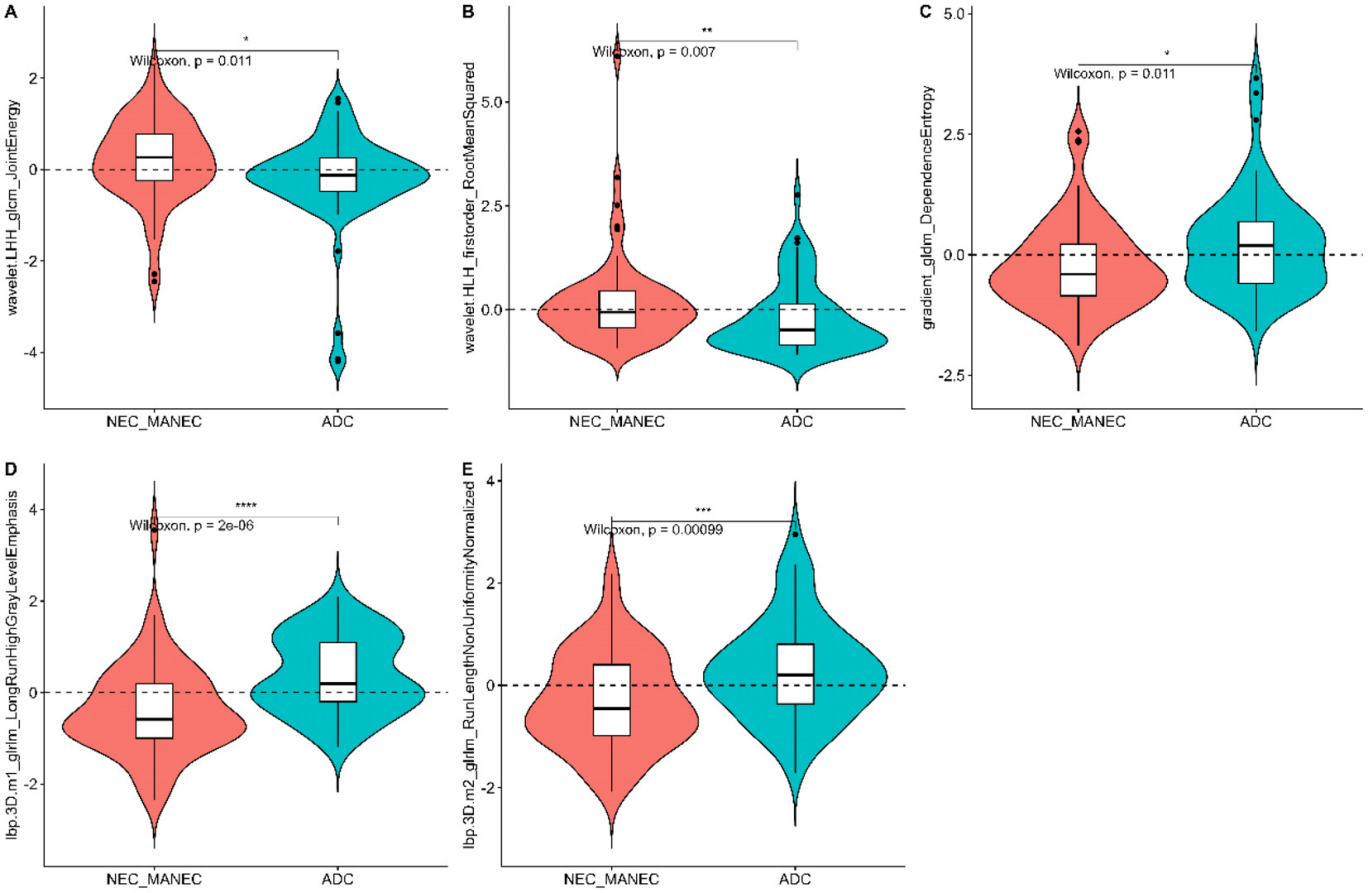
Violin plot of the five radiomics features in the radiomics model.

**Figure 4:**
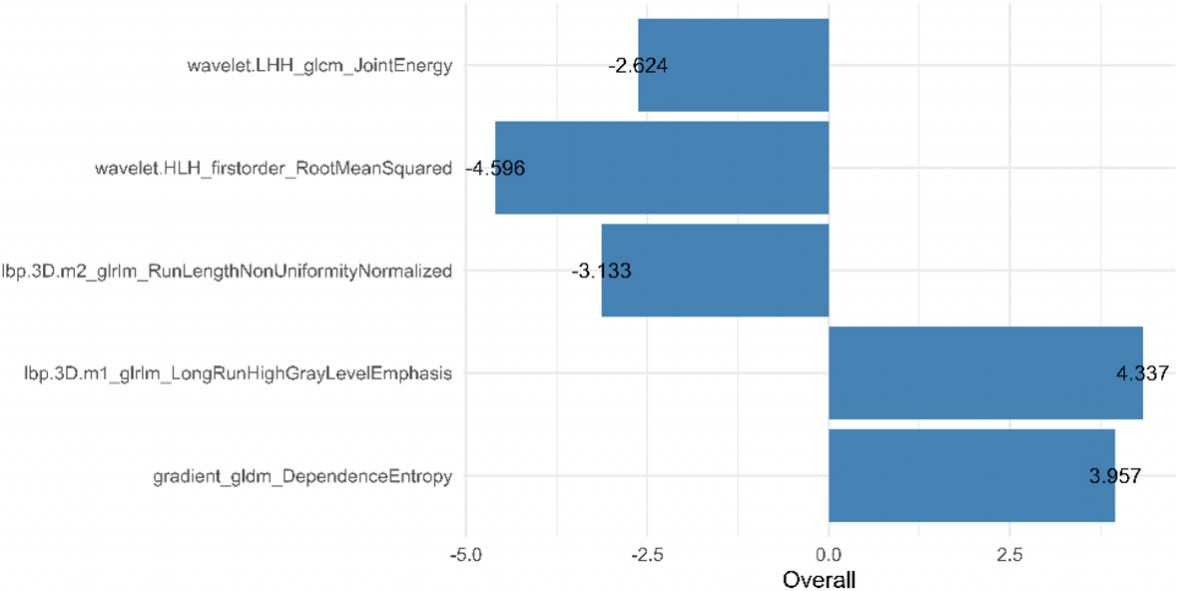
Weights of the five radiomics features in the radiomics model.

**Figure 5:**
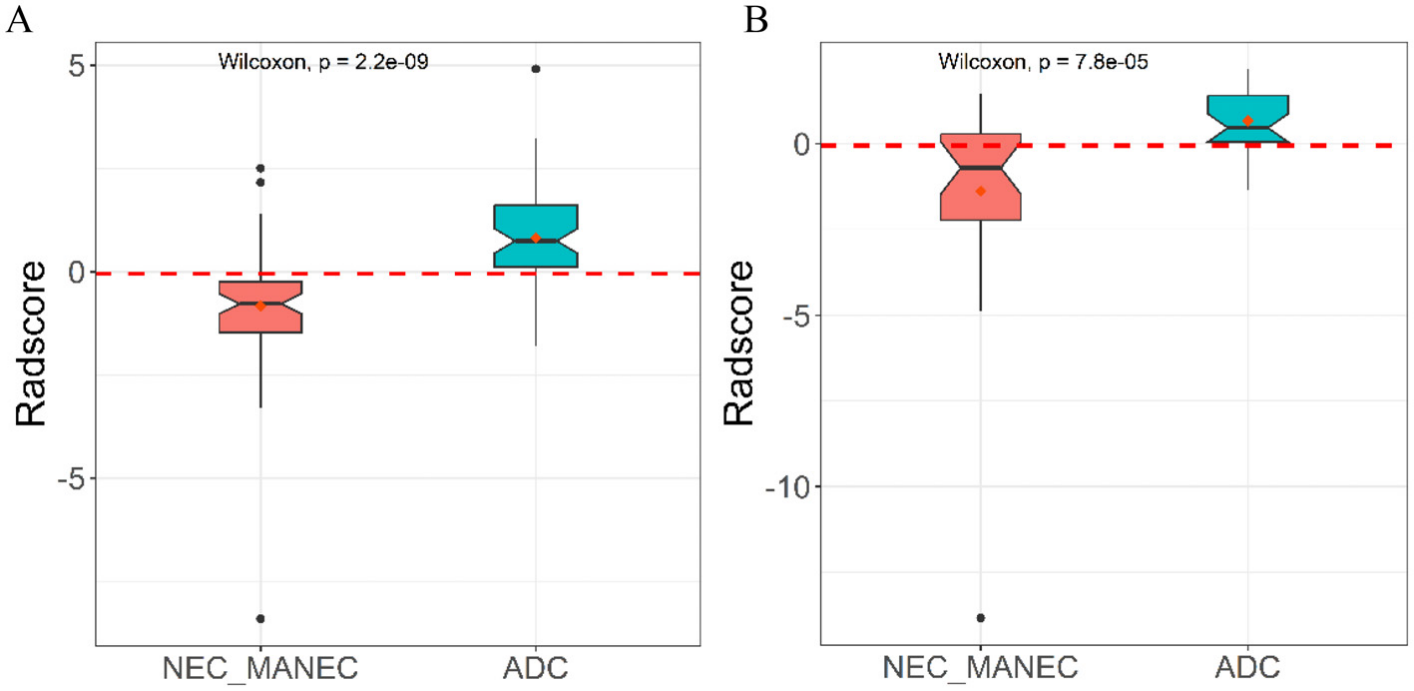
Distribution of radiomics model Rad-score in the training **(A)** and validation **(B)** cohort.

From the preprocessed original images, a total of 107 features were extracted, categorized as follows: 14 three-dimensional (3D) morphological features, 18 first-order statistical features, and 75 texture features. The texture features were further subdivided into various types based on different statistical matrices: 24 features from the gray level co-occurrence matrix (GLCM), 16 from the gray level run length matrix (GLRLM), 16 from the gray level size zone matrix (GLSZM), 14 from the gray level dependence matrix (GLDM), and 5 from the neighboring gray tone difference matrix (NGTDM). From the preprocessed filter image, 1395 features were extracted, totaling 1502 radiomics features. The feature selection process was rigorous and methodologically sound to ensure the robustness of the final radiomics model. Initially, through intraclass consistency analysis (ICC > 0.8), the feature set was narrowed down to 317. Subsequent univariate analysis further reduced this number to 133. Correlation analysis was then applied, retaining only 30 features that demonstrated minimal redundancy. LASSO regression helped in narrowing down these features to 17, and multivariable analysis finally selected 5 key features, including one first-order feature and four texture features (**Figure 3**). The first-order feature, root mean square, held the highest weight among them (**Figure 4**). These selected features were used to construct a radiomics model that outputs a Rad-score, depicted in **Figure 5**. The performance of this model in the training and validation cohort was remarkable, with AUC of 0.809 (95% CI, 0.731-0.887) and 0.802 (95% CI, 0.685-0.920), respectively.

### Model integration and comparison

**Figure 6:**
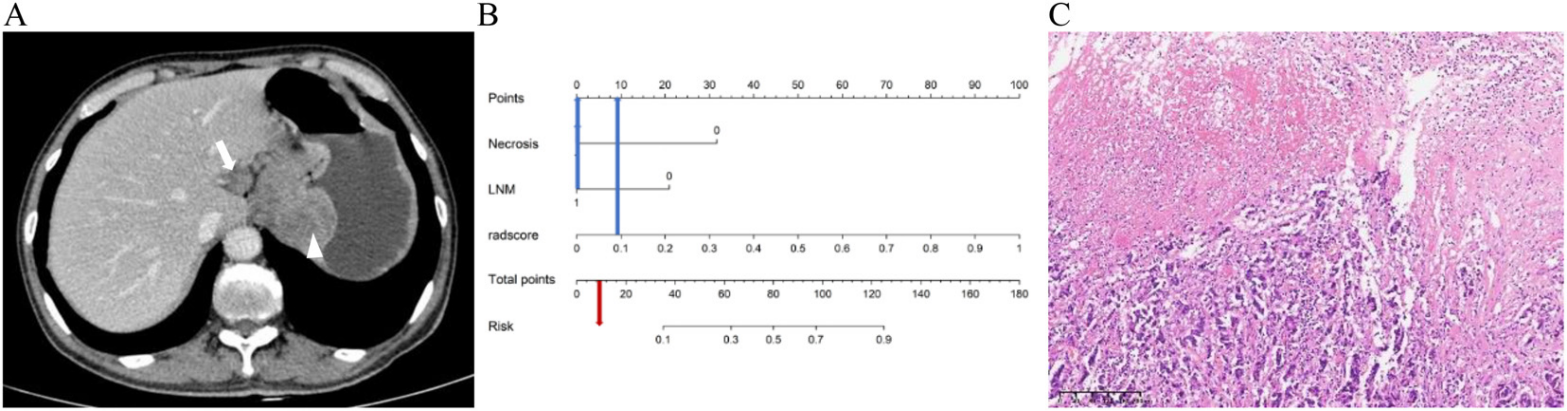
A 66-year-old male patient with g-NEC. **(A)** Axial CT image in the portal venous phase showed the thickening of the gastric wall in the upper part of the stomach with necrosis (arrowheads), and enlarged lymph node along the lesser curvature of the stomach (arrow) with a short diameter of 1.8cm. **(B)** The predicted risk value as illustrated by the Nomogram is below the critical point (0.36), hence leading to the diagnosis of g-(MA)NEC. **(C)** The pathological diagnosis is confirmed as g-NEC (HE, ×100).

**Figure 7:**
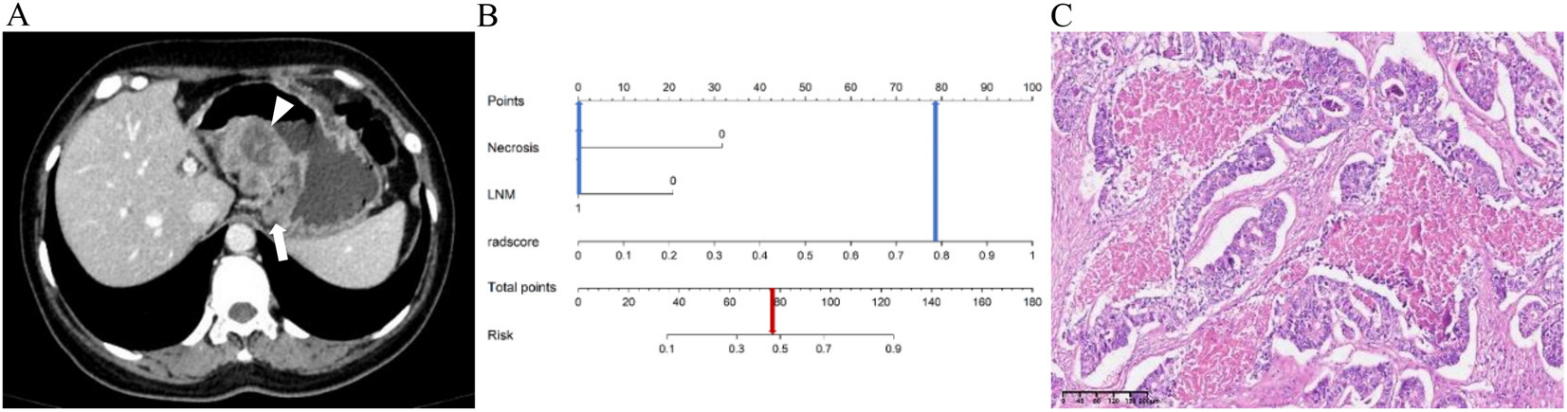
A 48-year-old female patient with g-ADC. **(A)** Axial CT image in the portal venous phase showed the thickening of the gastric wall in the middle part of the stomach with necrosis (arrowheads), and enlarged lymph node along the lesser curvature of the stomach (arrow) with a short diameter of 1.7cm. **(B)** The predicted value of Risk in the Nomogram exceeds the critical point (0.36), therefore the diagnosis is determined as g-ADC. **(C)** The pathological diagnosis is confirmed as g-ADC (HE, ×100).

The presence or absence of tumor necrosis, lymph node metastasis, and the Rad-score were identified as independent predictive factors for distinguishing between g-(MA)NEC and g-ADC, each demonstrating statistical significance (*P* < 0.05) as shown in **Table 2**. Utilizing these insights, a combined (clinical-radiomic) model was constructed, which significantly improved diagnostic accuracy. The effectiveness of this combined model was evident in its performance metrics, where it achieved AUC of 0.853 (95% CI, 0.786-0.919) in the training cohort and 0.812 (95% CI, 0.699-0.925) in the validation cohort. Additionally, a nomogram was created based on the combined model, as depicted in **Figures 6**, **7**. The diagnostic threshold was set at 0.36; predictions falling below this threshold suggest a diagnosis of g-(MA)NEC, while those above indicate g-ADC.

**Table 2:** Results of multifactor analysis on clinical model and combined model.

Variables	*β*	Clinical model OR (95%CI)	*P*	*β*	Combined model OR (95%CI)	*P*
Intercept	1.319			-1.377		
necrosis	-0.985	0.373 (0.151-0.840)	0.0209	-1.127	0.324 (0.118-0.831)	0.0228
cN	-1.551	0.212 (0.097-0.437)	<0.001	-1.079	0.340 (0.139-0.794)	0.0143
Rad-score				5.851	115.898 (25.541-627.644)	<0.001

**Figure 8:**
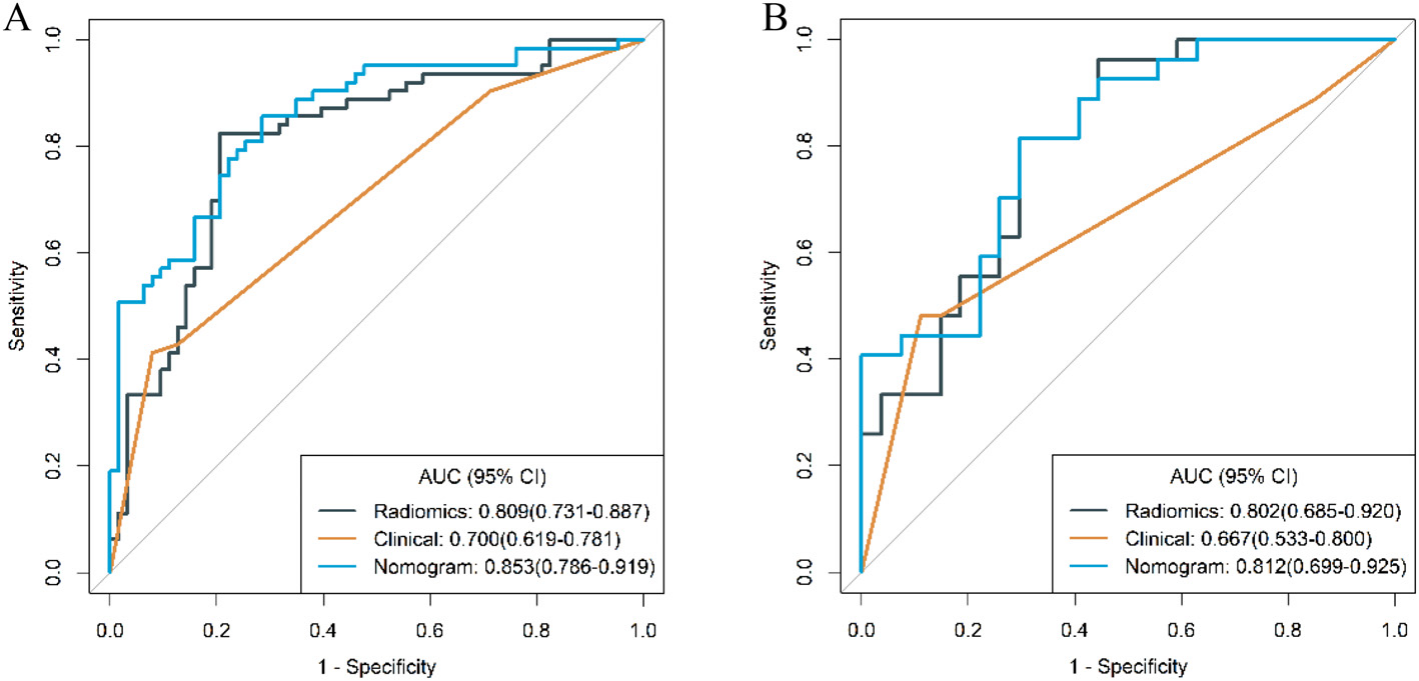
ROC curves of the three models in the training **(A)** and validation **(B)** cohort.

The ROC curves of the clinical model, radiomics model, and combined model are shown in **Figure 8**. **Table 3** shows ACC, SEN, SPE of the three models. The DeLong test revealed that in the training cohort, the combined model had a higher AUC compared to the clinical and radiomics models (*P*<0.001, *P*=0.031). In the validation cohort, the combined model had a higher AUC than the clinical model (*P*=0.019). However, there was no statistically significant difference in the AUC between the combined model and the radiomics model (*P*=0.734).

**Table 3:** The diagnostic performance of the three models in the training and validation cohort.

	Training cohort			Validation cohort		
Models	ACC (95%CI)	SEN (95%CI)	SPE (95%CI)	ACC (95%CI)	SEN (95%CI)	SPE (95%CI)
Clinical model	0.667(0.577-0.748)	0.413(0.180-0.548)	0.921(0.757-0.972)	0.685(0.544-0.805)	0.481(0.203-0.682)	0.889(0.578-1.000)
Radiomics model	0.810(0.730-0.874)	0.825(0.444-0.921)	0.794(0.444-0.857)	0.759(0.624-0.865)	0.815(0.370-1.000)	0.704(0.444-0.816)
Combined model	0.786(0.704-0.854)	0.857(0.682-0.952)	0.714(0.523-0.841)	0.722(0.584-0.835)	0.889(0.630-1.000)	0.556(0.369-0.815)

**Figure 9:**
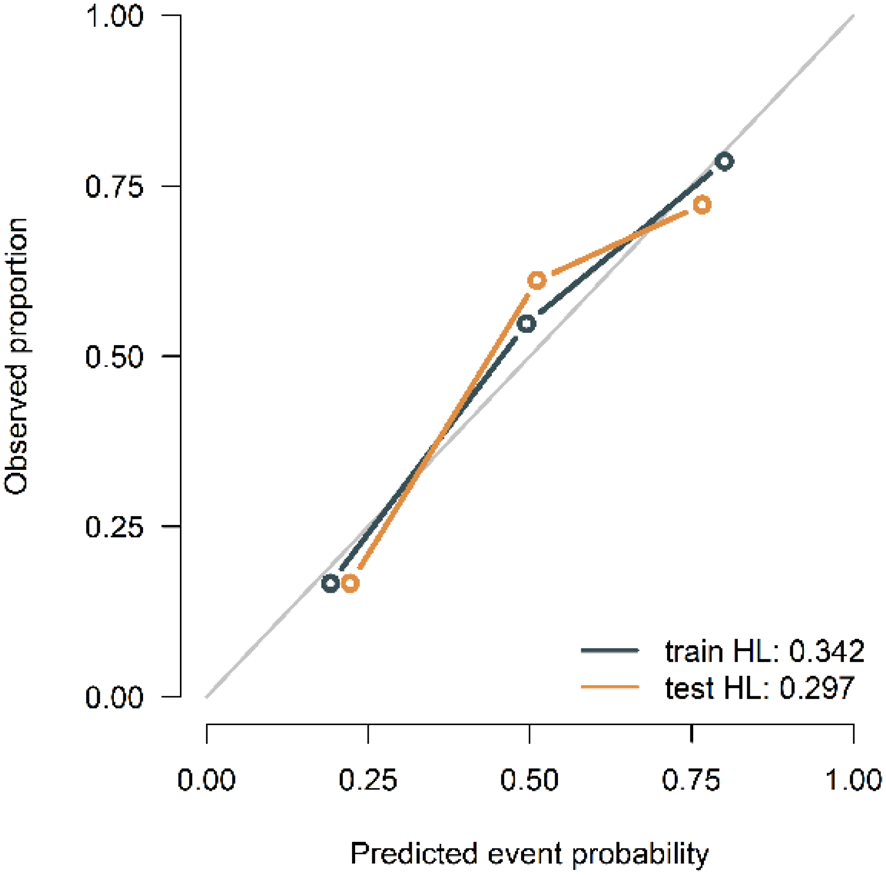
Calibration curves of the combined model in the training and validation cohort.

**Figure 10:**
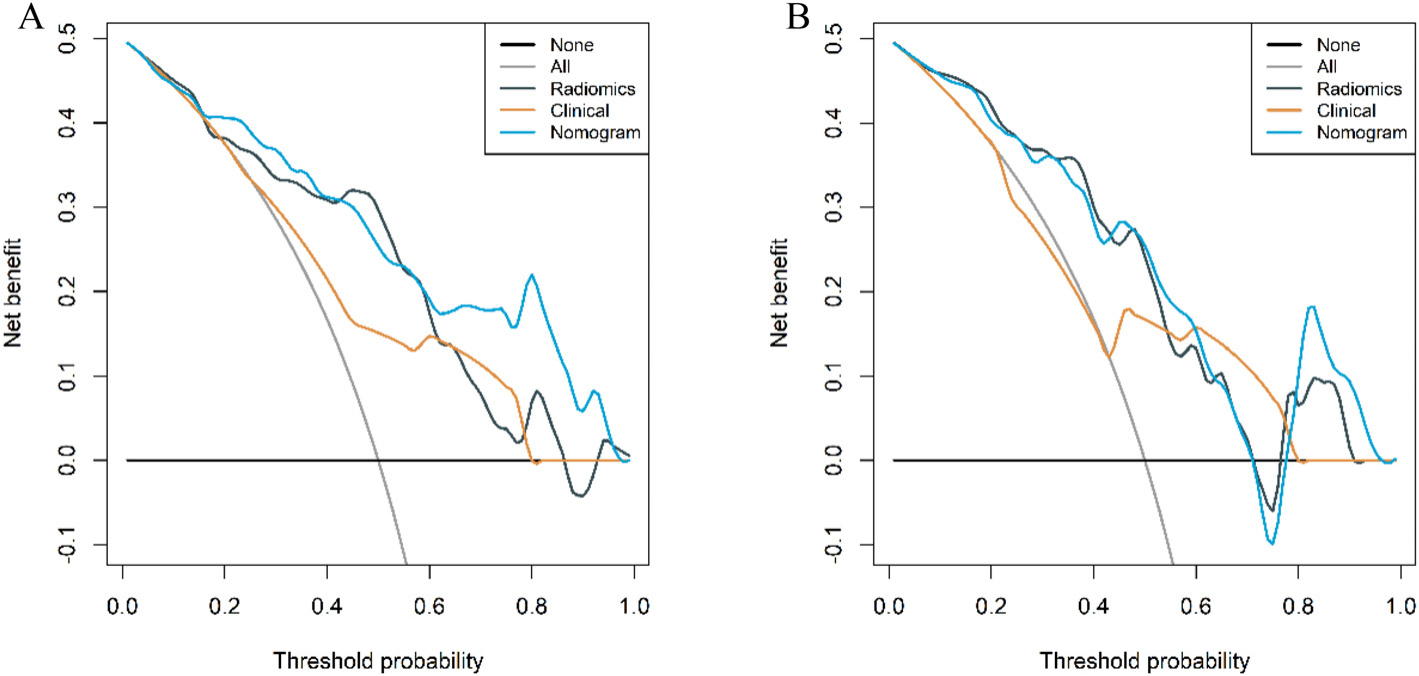
Decision curves of the three models in the training **(A)** and validation **(B)** cohort.

The calibration curves of the combined model in the training and validation cohort indicated strong concordance between the predicted values and the observed values (*P*=0.342, 0.297) (**Figure 9**). The DCA curves of the three models displayed are all higher than the two reference lines. Both the combined model and the radiomics model demonstrating greater net benefits than the clinical model (**Figure 10**).

### Differentiation of different pathologic subtypes

**Figure 11:**
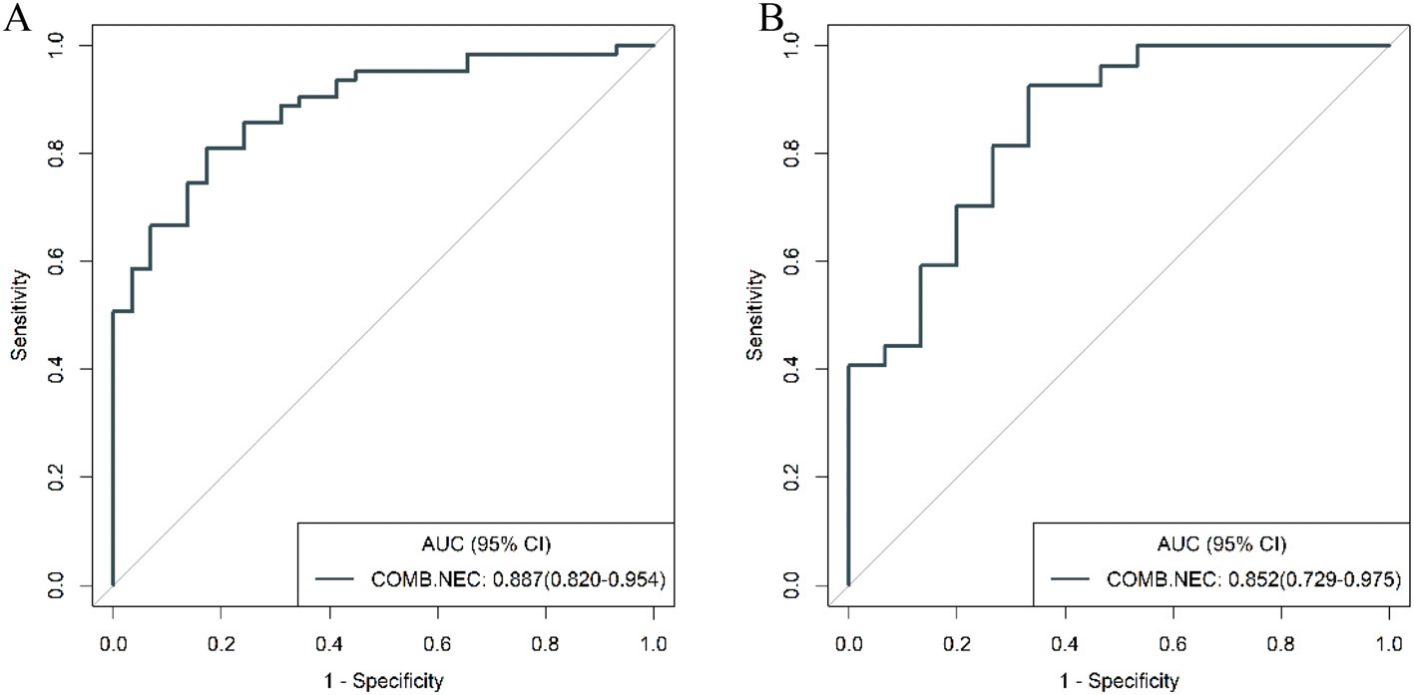
ROC curves for discriminating g-NEC and g-ADC of the combined model in the training **(A)** and validation **(B)** cohort.

**Figure 12:**
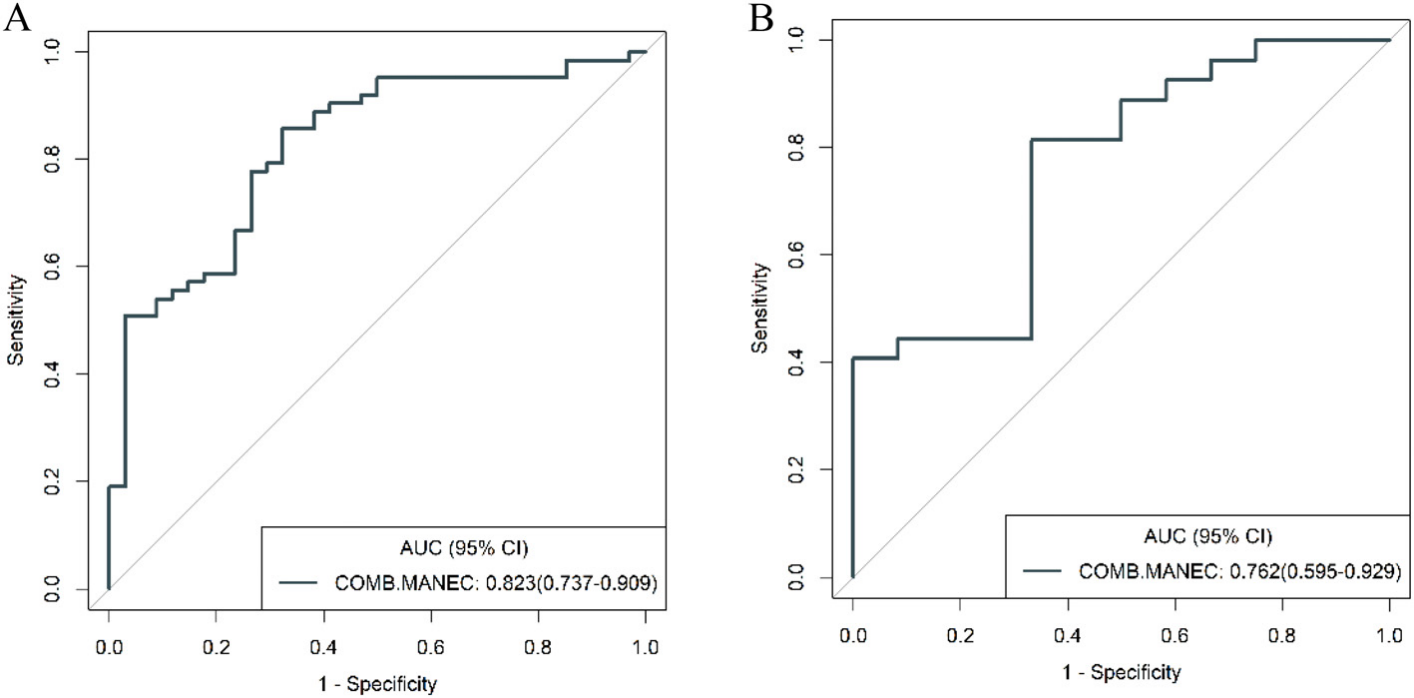
ROC curves for discriminating g-MANEC and g-ADC of the combined model in the training **(A)** and validation **(B)** cohort.

To further validate the diagnostic capabilities of the combined model, its performance was assessed using cases of g-NEC and g-ADC, as well as cases of g-MANEC versus g-ADC, which were included in the study cohort. The results of this validation process demonstrated high efficacy of the model in differentiating between these distinct gastric cancer subtypes. Specifically, the AUC for the model in distinguishing g-NEC from g-ADC were impressively high, recorded at 0.887 in the training cohort and 0.852 in the validation cohort. Similarly, for differentiating g-MANEC from g-ADC, the model achieved an AUC of 0.823 in the training cohort and 0.762 in the validation cohort, as illustrated in **Figures 11**, **12**.

## Discussion

g-(MA)NEC is characterized by a high malignancy grade, typically displaying aggressive growth patterns and poor prognosis compared to g-ADC. Due to these distinctions, treatment approaches for g-(MA)NEC differ from those for g-ADC. This study has developed and validated a predictive model based on traditional CT imaging and radiomic features, providing a non-invasive, effective preoperative diagnostic tool for differentiating g-(MA)NEC from g-ADC. The diagnostic performance of this model surpasses that of clinical model and holds promise in guiding the selection of preoperative treatment and surgical approaches for patients with g-(MA)NEC.

Currently, the clinical diagnosis of g-(MA)NEC relies heavily on pathological and immunohistochemical examinations. However, among the 90 g-(MA)NEC patients in this group, only 10 were definitively diagnosed preoperatively through gastroscopic biopsy (11.11%, 10/90). This low preoperative diagnostic rate may be attributed to the significant heterogeneity of gastric neuroendocrine carcinomas and the small tissue samples typically obtained via gastroscopy, which may not adequately reflect the pathological changes of the disease. Immunohistochemical markers such as chromogranin A (CgA) and synaptophysin (Syn) are crucial for confirming neuroendocrine differentiation of tumors and are indispensable in the diagnosis of neuroendocrine tumors. However, these tests are not routinely performed on gastroscopic biopsy samples, contributing to the low preoperative diagnostic rate for g-(MA)NEC.

This study assessed the clinical characteristics and traditional CT imaging features to evaluate their discriminative value between g-(MA)NEC and g-ADC. The findings confirmed that the presence of tumor necrosis and lymph node metastasis are independent predictive factors for differentiating these conditions. It was noted that g-(MA)NEC is more likely to exhibit tumor necrosis (33.3%) compared to g-ADC (11.1%). In research conducted by Feng et al. (19), the rate of necrosis in g-(MA)NEC was reported to be even higher, reaching 87.1%, and histopathological examinations frequently revealed extensive necrosis within g-(MA)NEC tumors (20). This propensity for necrosis may be attributed to the rapid proliferation of tumor cells and the immaturity of tumor vasculature, leading to ischemia and subsequent necrosis within the tumor mass.

The presence of lymph node metastasis has been validated in multiple studies as a differential factor between g-(MA)NEC and g-ADC (16, 21). The aggressive nature of g-(MA)NEC often leads to more frequent lymph node metastasis, underscoring the invasive characteristics of this tumor type. Univariable analysis in this study also highlighted significant differences between g-(MA)NEC and g-ADC in terms of tumor location, thickness, and △CTvp. G-(MA)NEC tumors were predominantly located in the upper region of the stomach (49/63, 77.8%), while g-ADC tumors were more commonly found in the lower stomach (37/63, 58.7%). This distribution aligns with previous research findings (2, 22, 23) and may be related to the typical distribution of enterochromaffin-like cells, which are abundant in the fundus and body of the stomach. The lower △CTvp observed in g-(MA)NEC may be associated with the more pronounced necrosis in these tumors.

Previous studies (16, 21) have indicated that the presence or absence of mucosal coverage on the surface of a tumor can serve as a distinguishing feature between g-NEC and g-ADC. G-(MA)NEC, for instance, is more likely to exhibit either a complete or partial mucosal layer over its surface or symmetric mucosal elevation at both ends of the tumor. This characteristic could be associated with the distribution of neuroendocrine cells within the mucosal layer rather than in the epithelial layer of the stomach. However, the findings of this current study reveal that both g-(MA)NEC and g-ADC show a mucosal coverage rate of 33.3%. This parity might be explained by the presence of peritumoral edema in cases of g-ADC, where the edematous areas can also exhibit enhanced mucosal layers, potentially leading to misinterpretation as positive tumor mucosal coverage. Given this, the evaluation of mucosal coverage appears to be highly subjective, and its diagnostic value remains questionable.

Radiomics analysis leverages high-throughput quantitative features to evaluate tumor heterogeneity more objectively and precisely than traditional imaging techniques. Existing studies have demonstrated that venous phase images effectively display the distribution of contrast agents within the tumor interstitium, which holds significant value for the differential diagnosis of gastric tumors (21, 24). In this study, 3D whole-tumor delineation based on venous phase CT images was performed, and a large set of extracted radiomic features underwent dimensionality reduction and stepwise selection. Ultimately, five radiomic features were retained. The retained features including First_Order_RootMeanSquared and GLCM_Joint energy, which measure image uniformity and reflect the spatial distribution of image intensities. Additional texture features from the GLRLM and the GLDM assess the correlation of image grayscale across planes or directions.

The study found lower uniformity and grayscale correlation in g-(MA)NEC compared to g-ADC, consistent with findings by Wang et al. (21). This may be associated with the higher necrosis rates and stronger heterogeneity in g-(MA)NEC, particularly in g-MANEC tumors, which contain varying components of adenocarcinoma and neuroendocrine carcinoma, thus further reducing image grayscale correlation (25). The radiomics model developed in this research achieved AUC of 0.809 and 0.802 in the training and validation cohorts, respectively, and its output Rad-score can be used for personalized quantitative analysis to more accurately assess tumor heterogeneity.

Radiomics combined with conventional CT imaging features and clinical features demonstrates great potential in tumor prediction and grading. Wang et al.’s (26) research findings reveal that a combined nomogram, which integrates radiomic signature based on plain CT images with clinical features, can effectively predict the pathologic grades of PNETs preoperatively with powerful predictive capability. Combining the presence or absence of tumor necrosis, lymph node metastasis, and the Rad-score from radiomic output, a combined model was constructed in this study. This model showed superior diagnostic efficacy with AUC of 0.853 and 0.812 in the training and validation cohort, respectively, outperforming the clinical model. To our knowledge, this is the first study to develop a combined model specifically for differentiating g-NEC and g-MANEC from g-ADC. Previous studies (21) have mainly focused on distinguishing neuroendocrine tumors from adenocarcinomas, without addressing the challenges posed by mixed tumors. We further validated our model’s performance in distinguishing g-NEC from g-ADC and g-MANEC from g-ADC separately. Subgroup analysis indicated that the combined model more effectively distinguished between g-NEC and g-ADC than between g-MANEC and g-ADC. This is likely because g-MANEC includes adenocarcinoma components, leading to overlapping features with g-ADC, which complicates differentiation. In this study, 21 out of 46 g-MANEC cases were predominantly adenocarcinoma (more than 50% adenocarcinoma components), and 10 had equal proportions of adenocarcinoma and neuroendocrine carcinoma components, accounting for 67.4% (31/46) of the g-MANEC cases. Nevertheless, the diagnostic efficacy of the combined model in distinguishing g-MANEC from g-ADC remained high at an AUC of 0.823, indicating good performance. This detailed subgroup analysis provides valuable insights into the model’s efficacy across different tumor subtypes, which has not been extensively explored in previous studies.

While this study provides insightful findings, it is important to acknowledge several inherent limitations. First, being a retrospective study conducted at a single center, there is potential for selection bias. This could influence the generalizability of the results to a broader population, as the sample may not fully represent the diversity of clinical scenarios seen in different geographic or institutional contexts. Second, g-(MA)NEC has a relatively low incidence rate, which naturally limits the sample size available for analysis. To minimize the risk of overfitting the model with an excessive number of features relative to the number of cases, the study deliberately retained a smaller number of radiomic features during the selection process. While this approach enhances the model’s robustness, it may also constrain the ability to capture the full spectrum of potentially informative features. Third, the CT images analyzed were obtained from different equipment, introducing potential confounding factors due to variations in imaging technology and protocols. Although image preprocessing was employed to standardize the images and reduce these confounding effects, some degree of variability inevitably remains, which could affect the precision of the radiomic analyses. To address these limitations and validate the findings, future research efforts should aim for a prospective, multi-center design involving a larger sample size. Such studies would not only help to confirm the validity and reliability of the results but also enhance their applicability in diverse clinical settings. A more extensive dataset, potentially gathered from multiple centers, would provide a more comprehensive understanding of the radiomic profiles associated with g-(MA)NEC and g-ADC, thereby facilitating the development of more accurate and universally applicable diagnostic models.

## Conclusion

A predictive model has been developed based on traditional CT imaging features and radiomics, offering a non-invasive and effective preoperative diagnostic method for distinguishing between g-(MA)NEC and g-ADC.

## Data Availability

The raw data supporting the conclusions of this article will be made available by the authors, without undue reservation.
